# pH-Sensitive Gold Nanorods for Non-Steroidal Anti-Inflammatory Drugs (NSAIDs) Delivery and DNA-Binding Studies

**DOI:** 10.3390/molecules28093780

**Published:** 2023-04-27

**Authors:** Eleni Zygouri, Vlasoula Bekiari, Georgios Malis, Nikos K. Karamanos, Christos Koutsakis, George Psomas, Vassilis Tangoulis

**Affiliations:** 1 Department of Chemistry, Laboratory of Inorganic Chemistry, University of Patras, 26504 Patras, Greece; eleni0503zig@hotmail.com; 2 Department of Crop Science, University of Patras, 30200 Messolonghi, Greece; bbekiari@upatras.gr; 3 Department of General and Inorganic Chemistry, Faculty of Chemistry, Aristotle University of Thessaloniki, 54124 Thessaloniki, Greece; georgosmalis@gmail.com; 4 Biochemistry, Biochemical Analysis & Matrix Pathobiology Research Group, Laboratory of Biochemistry, Department of Chemistry, University of Patras, 26504 Patras, Greece; n.k.karamanos@upatras.gr (N.K.K.); ckoutsakis@upatras.gr (C.K.); 5 Institute of Chemical Engineering Sciences (ICE-HT), Foundation for Research and Technology-Hellas (FORTH), 26504 Patras, Greece

**Keywords:** gold nanorods, PEGylation, NSAID, drug-release, interaction with DNA, affinity for albumins, cytotoxicity

## Abstract

A facile experimental protocol for the synthesis of poly(ethylene glycol)-modified (PEGylated) gold nanorods (AuNRs@PEG) is presented as well as an effective drug loading procedure using the non-steroidal anti-inflammatory drug (NSAID) naproxen (NAP). The interaction of AuNRs@PEG and drug-loaded AuNRs (AuNRs@PEG@NAP) with calf-thymus DNA was studied at a diverse temperature revealing different interaction modes; AuNRs@PEG may interact via groove-binding and AuNRs@PEG@NAP may intercalate to DNA-bases. The cleavage activity of the gold nanoparticles for supercoiled circular pBR322 plasmid DNA was studied by gel electrophoresis while their affinity for human and bovine serum albumins was also evaluated. Drug-release studies revealed a pH-sensitive behavior with a release up to a maximum of 24% and 33% NAP within the first 180 min at pH = 4.2 and 6.8, respectively. The cytotoxicity of AuNRs@PEG and AuNRs@PEG@NAP was evaluated against MCF-7 and MDA-MB-231 breast cancer cell lines. The development of AuNRs as an efficient non-steroidal anti-inflammatory drugs (NSAIDs) delivery system for chemotherapy is still in its infancy. The present work can shed light and inspire other research groups to work in this direction.

## 1. Introduction

Designing nano-platforms where properly functionalized inorganic nanoparticles (NPs) with specific capping ligands and/or bioactive agents are used to enhance biocompatibility, multifunctionality, and explicit targeting characteristics can provide a plethora of new opportunities for a wide range of applications [1,2,3,4,5]. For the case of gold NPs and nanorods (NRs), it has been found that surface functionalization with biomolecules, polymers, fluorophores, thiol-based molecules, and amino acids greatly enhance their properties concerning photothermal therapy [6,7,8,9], cancer theranostics [6], drug/gene delivery [10,11] as well as sensing/imaging purposes [10,11]. For the synthesis of Au NPs/NRs, the seed-growth technique is widely used while other techniques such as chemical vapor deposition, microwave, electrochemical, and pyrolysis, to name a few, are also presented [10,11]. Concerning the surface functionalization of the Au NPs/NRs, the experimental procedures used involve: (a) effective coating with polymers; (b) immobilization of capping ligands with covalent bonds or bio-affinity; (c) physical sorption methods; (d) green chemistry protocols; and € chemical reduction methods. The goal of these surface modifications is the improvement of the colloidal stability as well as the biocompatibility avoiding aggregation phenomena as well as sedimentation [9,10,12,13,14].

Due to the large surface area, Au NPs/NRs present exciting features as nanoplatforms where therapeutic agents such as drugs can be efficiently loaded or conjugated. While the development of Au NPs/NRs as efficient non-steroidal anti-inflammatory drugs (NSAIDs) delivery systems for chemotherapy purposes is still in its infancy, several studies are focused on Au NPs for the effective delivery/release of anticancer drugs. In the latter case, certain issues concerning toxicity and biocompatibility as well as the possible clinical application are under scrutiny [12,15,16,17,18,19]. For instance, Au NPs were designed for intracellular delivery and the pH-triggered release of anticancer drug doxorubicin (DOX) functionalized with thiolated poly(ethylene glycol) (PEG) which was further covalently linked to a polyamidonamine G4 dendrimer [12].

In this study, a nanoplatform was developed for NSAID naproxen (NAP) drug delivery and release based on PEGylated AuNRs. The modification of the surface with PEG was mandatory to ensure colloidal stability by increasing the interparticle repulsion of a coulombic nature as well as the biocompatibility of the nanoplatforms [20]. It has been shown that the PEGylation of AuNPs increases their accumulation near tumor sites (in vivo) due to the improved mobility of AuNPs in the bloodstream [21]. Drug release studies were performed to evaluate whether targeted drug delivery is pH-sensitive. As a means to evaluate the potential biological targets of AuNRs@PEG and AuNRs@PEG@NAP and to investigate possible mechanisms of action, the interaction of them with biomacromolecules was focused on the investigation of: (i) their interaction with calf-thymus (CT) DNA by UV-vis spectroscopy and via their ability to displace a typical intercalator (i.e., ethidium bromide, EB) from its DNA-adduct explored by fluorescence emission spectroscopy, (ii) their ability to cleave supercoiled circular pBR322 plasmid DNA (pDNA) studied by gel electrophoresis, and (iii) their affinity for human serum albumin (HSA) and bovine serum albumin (BSA) evaluated by fluorescence emission spectroscopy. Furthermore, the cytotoxicity of AuNRs@PEG and AuNRs@PEG@NAP was evaluated against two breast cancer cell lines: the low metastatic MCF-7 cells, and the more aggressive MDA-MB-231 cells.

## 2. Results and Discussion

### 2.1. General Synthetic Aspects

For the synthesis of the AuNRs, a seed-mediated growth method was followed using binary surfactant mixtures of CTAB/NaOL with some modifications [22]. The absorption spectrum of AuNRs revealed two absorption peaks at ca. 517 nm (transverse SPR, t-SPR) and at ~1025 nm (longitudinal SPR, l-SPR). The peak position of the l-SPR is related to the aspect ratio of the AuNRs according to Equation (1) [23] where R is the aspect ratio and the calculated value is AR = 6.3.

(1) $$\lambda_{max}\left(nm\right)=95R+420$$

**Figure 1:**
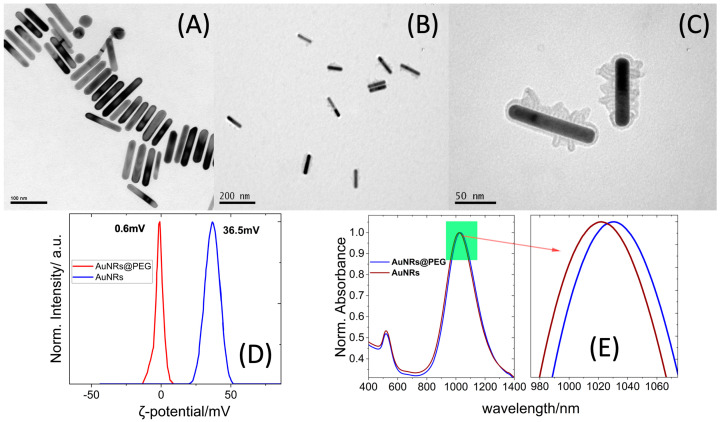
(**A**) TEM images of AuNRs. (**B**,**C**) TEM images of AuNRs@PEG. (**D**) ζ-potential measurements of AuNRs and AuNRs@PEG. (**E**) UV-vis-NIR absorption spectra of AuNRs and AuNRs@PEG.

The TEM images revealed rod-shaped gold NPs with a length of 90.0 ± 8.0 nm and width of 15.3 ± 3.4 nm and an aspect ratio of AR~6.1 close to the value obtained from the UV-vis/near-IR measurements (Figure 1 and Figure S1).

The effective PEGylation of the AuNRs was further supported by the red shift (12 nm) of the l-SPR band of the absorption spectrum of the AuNRs@PEG dispersions [24] as well as the decrease of the ζ-potential from high positive values of 36.7 mV (CTAB-covered AuNR s) to almost close to zero values (~0.6 mV) for AuNRs@PEG [24]_._

**Figure 2:**
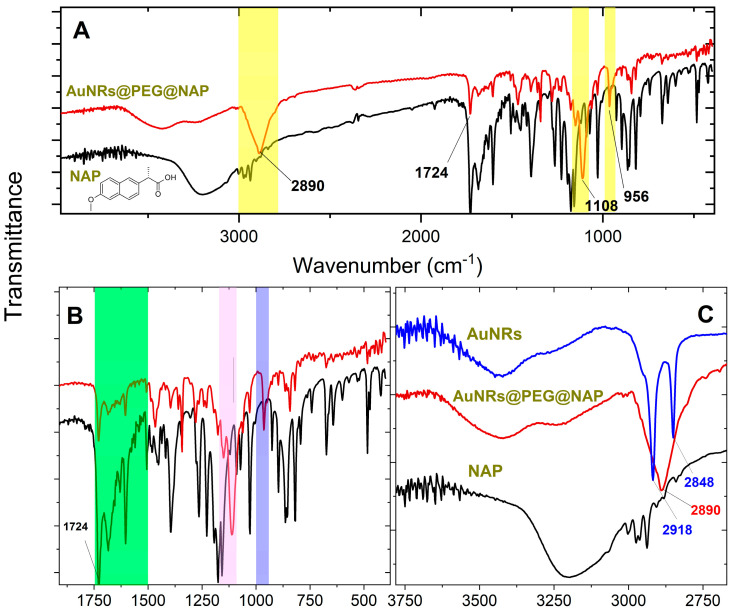
(**A**) IR spectra of AuNRs@PEG@NAP (red line) and NAP (black line) where three distinctive areas (highlighted with yellow) present bands related to the PEG polymer (see text for details). (**B**) IR spectra of AuNRs@PEG@NAP (red line) and NAP (black line) in the region 1800–400 cm^−1^) where two areas (highlighted with purple and violet) present bands related to the PEG polymer and another one highlighted with green confirms the effective loading of NAP (see text for details). (**C**) IR spectra of originally CTAB synthesized AuNRs (blue line), AuNRs@PEG@NAP (red line), and NAP (black line) in the region 3800–2700 cm^−1^ (see text for details).

Another confirmation of the effective PEGylation of the AuNR’s surface is based on FT-IR spectroscopy. The IR spectra of the AuNRs@PEG@NAP are shown in Figure 2 where three distinctive areas (highlighted with yellow) present bands related to the PEG polymer. More explicitly: (i) the stretching and vibration band of the C-O-C ether is located at 1108 cm^−1^ (violet region in Figure 2B) and (ii) the out-on-plane vibration band of CH is shown at 956 cm^−1^ (purple region in Figure 2B) and at 2890 cm^−1^ (Figure 2C). The effective ligand exchange between CTAB and mPEG-SH during the PEGylation procedure is also confirmed by the extinction of the two characteristic bands at 2918 and 2848 cm^−1^ belonging to the asymmetric bands of CH_2_/CH_3_ groups of CTAB (Figure 2C) [25]. Furthermore, the effective loading of NAP in AuNRs@PEG@NAP is also confirmed in the IR spectrum by the characteristic stretching vibration of the carboxylic group at 1724 cm^−1^ as well as the breathing vibrations of the aromatic rings resolved in the region highlighted with green in Figure 2B. It should be pointed out that using thiol-based PEG derivatives, the ligand exchange reaction is greatly improved leading to the successful elimination of the CTAB and highly efficient PEGylation.

### 2.2. Drug Release Studies

**Figure 3:**
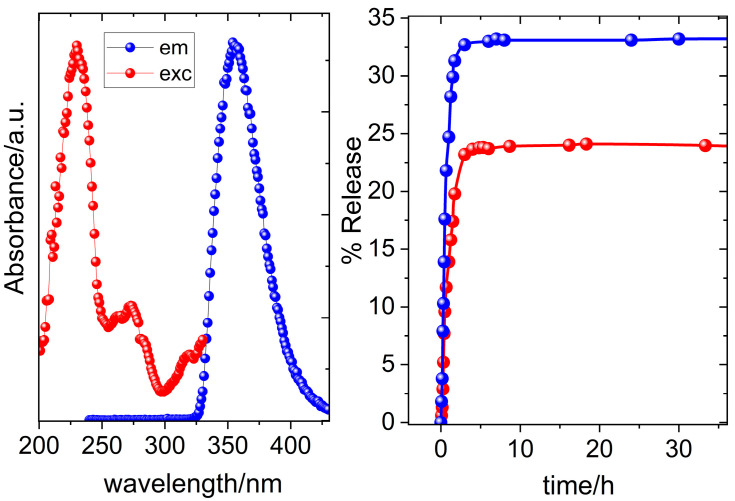
(**left**) Excitation spectrum of AuNRs@PEG@NAP at λ_em_ = 359 nm (red solid spheres) and emission spectrum of AuNRs@PEG@NAP at λ_exc_ = 233 nm (blue solid spheres); (**right**) release of NAP at pH = 6.8 (blue solid spheres) and at pH = 4.2 (red solid spheres).

The emission spectra of a solution of free NAP (red line) and the suspension of AuNRs@PEG@NAP (green line) are presented in Figure S2 while no emission was detected for AuNRs@PEG. A comparison between the spectra of the solution of free NAP (λ_max,em_ = 357 nm) and AuNRs@PEG@NAP (λ_max,em_ = 359 nm) reveals a significant broadening and a slight red shift in λ_max,em_ for the latter one. Both of these features can be attributed to changes in the chemical environment surrounding the drug molecule due to its location on the surface of AuNRs@PEG@NAP suggesting a successful loading of the drug. It was possible to calculate the percentage of the drug loading from the calibration curve of the emission intensity of naproxen at 357 nm and it was found to be 112.5 mg/g (Figure S3). The excitation and emission spectra of an aqueous suspension of AuNRs@PEG@NAP are presented in Figure 3. Due to the fluorescence properties of NAP, the successive loading of the drug on the pegylated surfaces of gold NRs was confirmed by using fluorescence presenting a maximum excitation and emission at 233 nm and 359 nm, respectively, with a large stoke shift of 123 nm [26,27].

In order to check whether targeted drug delivery is pH-sensitive, two representative pH values (4.2, 6.8) were chosen having in mind that the acidic conditions (pH = 4.2) are typical of inflamed tissues. At pH 4.2, the release profile indicates a release up to a maximum of 24% NAP within the first 180 min, while a higher percentage of NAP release (33%) was observed at pH = 6.8 for the same time value (Figure 3). The pronounced burst effect experienced in the first 3 h is possibly related to the desorption of a percentage of the drug close to the surface and its immediate release upon contact with the release medium since PEG is water soluble [28,29]. After the first 3 h, an extremely slow release rate was monitored for both pH values directly related to a change of the release mechanism to interlayer diffusion of protected NAP molecules among the stacking of PEG polymers [30,31,32].

Since this is the first example of an AuNR nano-platform loaded with NSAID drugs, there are no direct comparisons with other AuNR drug carriers concerning the loading/release mechanisms. Nevertheless, the release profiles are similar to other PEGylated ferrite NPs, polymeric capsules and polypeptide vesicles [30,31,32].

For the following biomacromolecule-binding studies, the aqueous colloidal dispersions of AuNRs@PEG@NAP were prepared in pH = 4.0 and the measurements were performed in freshly prepared samples and in a time duration of 1–2 h to ensure that the loaded NAP on the surface of the gold is not released.

### 2.3. Interaction of AuNRs with CT DNA

DNA is usually a potential biomolecular target for diverse drugs such antibacterial, antiviral, and anticancer agents [33]. The investigation of the interaction of potentially bioactive compounds with DNA is often employed either complimentary to cytotoxic studies or in order to obtain an insight of possible mechanisms or applications. In general, compounds may interact with DNA in three fashions, i.e., via covalent binding or non-covalent interactions or cleavage of the DNA helix [34]. The interaction of AuNRs@PEG and AuNRs@PEG@NAP with CT DNA was investigated directly by UV-vis spectroscopy indirectly by evaluating their ability to displace EB from the EB–DNA adduct.

**Figure 4:**
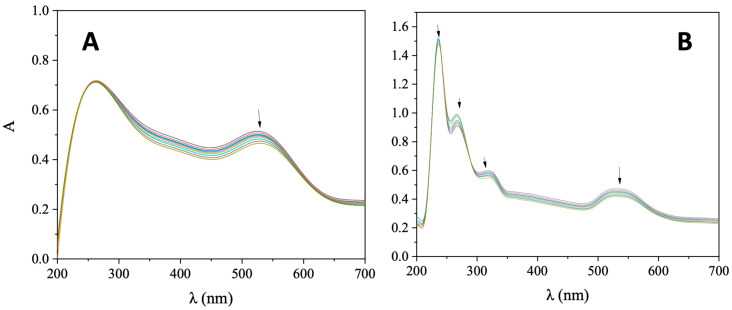
UV-vis spectra of (**A**) AuNRs@PEG and (**B**) AuNRs@PEG@NAP (0.25 mg/mL) in buffer solution (100 mM CH_3_COOH and 25 mM CH_3_COONa, pH = 4) in the presence of increasing amounts of CT DNA. All the spectra were recorded at 18 °C. The arrows show the changes upon the addition of CT DNA solution.

UV-vis spectroscopic titrations were employed initially to assess the interaction between CT DNA and AuNRs@PEG and AuNRs@PEG@NAP, in order to gain information on the mechanism and the intensity of this interaction. During such UV-vis spectroscopic titration investigations, the UV-vis spectra of AuNRs@PEG and AuNRs@PEG@NAP were recorded in the presence of increasing amounts of CT DNA. More specifically, in the UV-vis spectrum of AuNRs@PEG, the band observed at 525 nm (Figure 4A) exhibited in the presence of CT a slight hypochromism accompanied by a slight red shift (Table 1). In the UV-vis spectrum of AuNRs@PEG@NAP, two basic bands were observed (Figure 4B); band I at 530 nm which may be attributed to the presence of a Au-nanorod since it was also found for AuNRs@PEG and band II at 319 nm which may be assigned to the presence of naproxen, as also found in reported naproxen complexes [35,36,37,38,39]. In the presence of CT DNA, these two bands showed a slight hypochromism up to 10% followed by a slight bathochromism (Table 1). Such features may indicate the interaction of AuNRs@PEG and AuNRs@PEG@NAP with CT DNA, although the possible interaction mode may not be concluded safely.

The binding constants of AuNRs@PEG and AuNRs@PEG@NAP with CT DNA (K_b_) were calculated with the Wolfe–Shimer equation (Equation (S1)) [40] and the corresponding plots [DNA]/(ε_A_ − ε_f_) versus [DNA] (Figure S4). At the temperature of 18 °C, AuNRs@PEG@NAP presents lower K_b_ than AuNRs@PEG but higher K_b_ (almost two times) than free naproxen. This may suggest that the incorporation of naproxen in the AuNRs@PEG may lead to a tighter interaction with CT DNA than the free NAP. A comparison of the DNA-binding constant of AuNRs@PEG@NAP with the reported metal–naproxen complexes may reveal that AuNRs@PEG@NAP is a slightly tighter DNA-binder than the Co(II) [37], Cu(II) [38], and most Mn(II) [35,36] complexes of naproxen, and similar to Sn(IV) [41] and Ag(I) [42] complexes, while the polyethyleneimine-functionalized carbon nanotube hosting naproxen [43], the water-soluble silica hybrid spin-crossover nanoparticle loaded with naproxen [44], and the reported Ni(II)–naproxen complexes present higher K_b_ values [39] than AuNRs@PEG@NAP.

In order to obtain deeper information regarding the possible interaction forces developed between AuNRs@PEG and AuNRs@PEG@NAP with CT DNA, the UV-vis spectroscopic titration studies in the presence of CT DNA were performed for three different temperatures (291 K, 300 K, and 310 K) and the corresponding K_b_ values were also determined (Table 2). It may be noted that the increase of temperature results in a lower K_b_ value for AuNRs@PEG and a significantly increased K_b_ value for AuNRs@PEG@NAP. Such a trend may suggest different DNA interaction modes for AuNRs@PEG and AuNRs@PEG@NAP.

**Figure 5:**
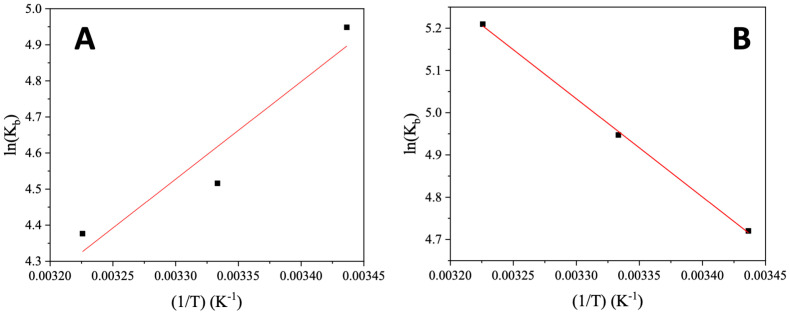
Van’t Hoff plots for the interaction of CT DNA with (**A**) AuNRs@PEG and (**B**) AuNRs@PEG@NAP.

Among the interaction forces developed between a bioactive compound and a biomolecule, the most common ones are hydrophobic forces, electrostatic interactions, van der Waals interactions, and hydrogen bonds [45,46]. The enthalpy change (ΔH) and the entropy change (ΔS) may be calculated from the Van ’t Hoff equation (Equation (S2)) and the plots of ln(K_b_) versus (1/T), where –ΔH/R is the slope of the fitting line and ΔS/R is the intercept (R is the universal gas constant) (Figure 5). In addition, ΔG may be obtained from the Gibbs–Helmholtz equation (Equation (S3)).

Three different combinations of the enthalpy change (ΔH) and the entropy change (ΔS) have been reported in the literature which are related to the development of different types of interaction between the compound and the biomacromolecule: (a) in the case of ΔH > 0 and ΔS > 0, hydrophobic forces are developed, (b) the combination ΔH < 0 and ΔS < 0 may exist in the case of van der Waals interactions, and hydrogen bonds and (c) electrostatic interactions may lead to ΔH < 0 and ΔS > 0 [46,47]. From the plots ln(K_b_) versus (1/T) for AuNRs@PEG and AuNRs@PEG@NAP (Figure 5), the corresponding ΔH and ΔS values were determined (Table 2). For AuNRs@PEG, both values of ΔH and ΔS are negative revealing the development of van der Waals interactions and hydrogen bonds between AuNRs@PEG and CT DNA which may subsequently indicate the existence of external interactions (groove-binding) with DNA [48,49,50]. In the case of AuNRs@PEG@NAP, both values of ΔH and ΔS are positive suggesting the existence of hydrophobic forces between AuNRs@PEG@NAP and CT DNA, stabilized by π–π stacking interactions which may be explained by the existence of an intercalation [48]. The negative ΔG values for both AuNRs@PEG and AuNRs@PEG@NAP may show that their interaction with CT DNA is spontaneous [46,48,50,51].

**Figure 6:**
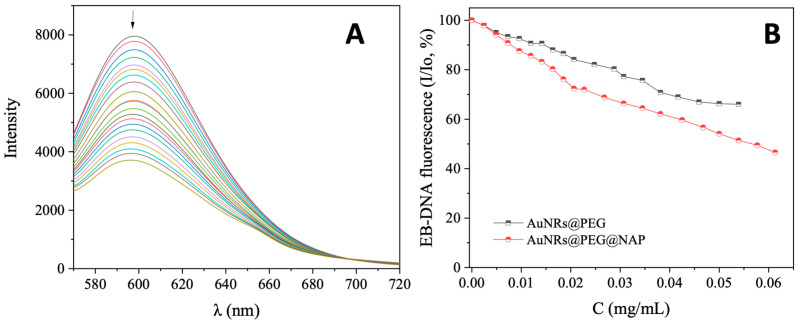
(**A**) Fluorescence emission spectra (λ_excitation_ = 540 nm) for EB–DNA adduct ([EB] = 20 μM, [DNA] = 26 μM) in buffer solution (150 mM NaCl and 15 mM trisodium citrate at pH = 7.0) in the absence and presence of increasing amounts of AuNRs@PEG@NAP. The arrow shows the changes of intensity upon increasing amounts of AuNRs@PEG@NAP. (**B**) Plot of EB–DNA relative fluorescence intensity at λ_emission,max_ = 592 nm (I/Io, %) versus *r* (*r* = [complex]/[DNA]) for AuNRs@PEG and AuNRs@PEG@NAP (up to 66.0% of the initial EB–DNA fluorescence intensity for AuNRs@PEG (black line), and 46.6% for AuNRs@PEg@NAP (red line).

EB is a typical DNA intercalator with a known DNA-binding constant (K_b_ = 1.23 (±0.07) × 10^5^ M^−1^) [52]. An indication of its DNA intercalation is the appearance of a strong fluorescence emission band at 592 nm, upon excitation of its solution at 540 nm [53]. A DNA intercalator may displace EB from the EB–DNA adduct resulting in a quenching of the EB–DNA fluorescence emission band. In the present case, the EB–DNA adduct was prepared by the 1 h pre-treatment of a solution containing 20 µM EB and 26 µM CT DNA. The addition of AuNRs@PEG and AuNRs@PEG@NAP in increasing amounts results in a quenching of the EB–DNA band (Figure 6 and Figure S5) which was more intense in the presence of AuNRs@PEG@NAP (up to 46.6% of the initial EB–DNA fluorescence) (Table 3). This quenching is obviously a result from the displacement of EB from the EB–DNA due to the competition with AuNRs@PEG@NAP for the DNA intercalation sites.

The Stern–Volmer plots (Figure S6) show that the quenching of the EB–DNA fluorescence emission is in good agreement (R^2^~0.99) with the linear Stern–Volmer equation (Equation (S4)), which suggests that the quenching may be assigned to the EB-displacing ability of AuNRs@PEG and especially AuNRs@PEG@NAP. The K_SV_ constants (Table 3) of AuNRs@PEG and AuNRs@PEG@NAP were calculated by the Stern–Volmer equation and the Stern–Volmer plots (Figure S6). The EB–DNA system has a fluorescence lifetime (τ_o_) of 23 ns [54] which is applied for the determination of the corresponding EB–DNA quenching constants (k_q_) with Equation (S5) [53]. The k_q_ values of the AuNRs@PEG and AuNRs@PEG@NAP (Table 3) may suggest the existence of a static quenching mechanism for the EB–DNA fluorescence [55]. A direct comparison of the constants of AuNRs@PEG@NAP with those of the reported metal–naproxen complexes may not be achieved due to their expression in the molar scale. Given the molecular mass of NAP (230.2 g/mol), the corresponding constants could be expressed in mg/mL and are much higher than those of AuNRs@PEG@NAP. In addition, the constant of AuNRs@PEG@NAP could be considered comparable with those of a previously reported carbon nanotube and spin-crossover nanoparticle loaded with naproxen [43,44].

### 2.4. Interaction of the AuNRs with pBR322 Plasmid DNA

**Figure 7:**
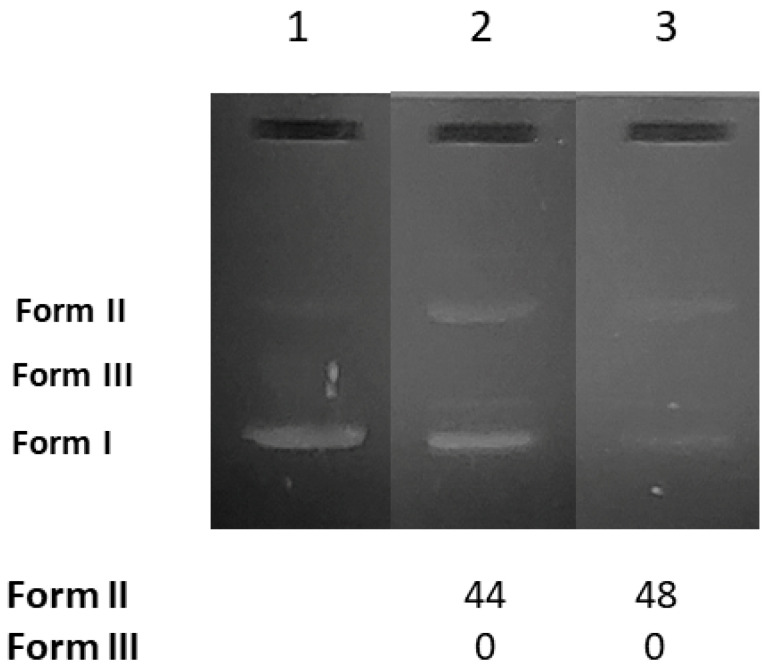
Agarose gel electrophoretic pattern of EB-stained plasmid DNA (pBR322 plasmid DNA) with AuNRs@PEG and AuNRs@PEG@NAP at 0.25 mg/mL. Top: Gel electrophoreses pictures: Lane 1: DNA; Lane 2: DNA + AuNRs@PEG; and Lane 3: DNA + HLHΛAuNRs@PEG@NAP. Bottom: Calculation of the % conversion to ss and ds damage. DNA forms: Form I = supercoiled, Form II = relaxed, and Form III = linear plasmid DNA.

As a next step to the studies with CT DNA, the cleavage of DNA was investigated in order to evaluate their potential nuclease-like ability. The efficiency of the cleavage of the pBR322 plasmid DNA (pDNA) with AuNRs@PEG and AuNRs@PEG@NAP was visualized and measured by the gel electrophoresis technique. The supercoiled pDNA in an agarose gel during electrophoresis is shown as Form I (Figure 7, Lane 1). In general, a potential cleaving activity by the AuNRs@PEG and AuNRs@PEG@NAP may be revealed as single-stranded nicks (ss) in the supercoiled DNA leading to the formation of the relaxed circular DNA (Form II) and/or double-stranded nicks (ds) forming linear DNA (Form III) and are calculated with Equations (S6) and (S7). All experiments were carried out in duplicate.

AuNRs@PEG and AuNRs@PEG@NAP were mixed with pDNA (Tris buffer solution, 25 μΜ, pH = 6.8). After incubation of the components, the pDNA was analyzed by gel electrophoresis on 1% agarose stained with EB. As shown in Figure 7, both AuNRs@PEG and AuNRs@PEG@NAP may induce single-stranded nicks (Form II) up to 48% (Figure 7, Lane 3, for AuNRs@PEG@NAP). In total, the AuNRs presented a rather moderate cleavage activity at a rather high concentration (0.25 mg/mL) with AuNRs@PEG@NAP being marginally more active (48%) than AuNRs@PEG (44%).

### 2.5. Interaction of the AuNRs with Albumins

**Figure 8:**
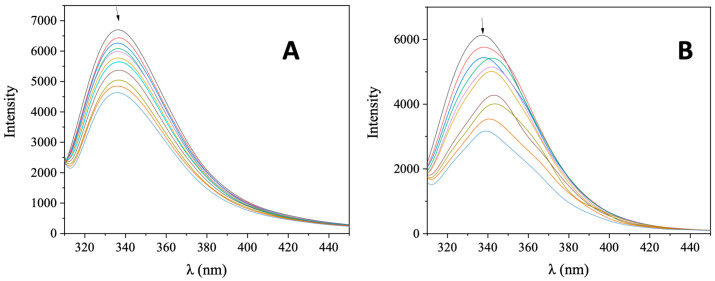
Fluorescence emission spectra (λ_excitation_ = 295 nm) for ΒSA ([ΒSA] = 3 μM) in buffer solution (150 mM NaCl and 15 mM trisodium citrate at pH 7.0) in the absence and presence of increasing amounts of (**A**) AuNRs@PEG and (**B**) AuNRs@PEG@NAP. The arrow shows the changes of intensity upon increasing amounts of AuNRs@PEG and AuNRs@PEG@NAP.

**Figure 9:**
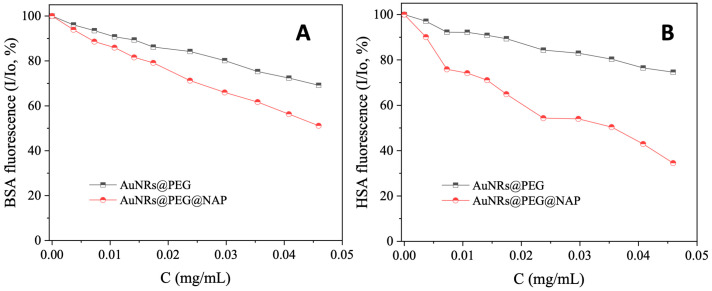
(**A**) Plot of relative fluorescence emission intensity of BSA at λ_emission,max_ = 339 nm (I/Io, %) versus the concentration of AuNRs@PEG and AuNRs@PEG@NAP (up to 69.2% of the initial BSA fluorescence for AuNRs@PEG (black line), and 51.1% for AuNRs@PEG@NAP (red line). (**B**) Plot of relative fluorescence emission intensity of HSA at λ_emission,max_ = 337 nm (I/Io, %) versus the concentration of AuNRs@PEG and AuNRs@PEG@NAP (up to 74.6% of the initial HSA fluorescence for AuNRs@PEG (black line), and 34.5% for AuNRs@PEG@NAP (red line).

Serum albumin (SA) is one of the most significant proteins in plasma since it contributes to the transportation of metal ions, drugs, and small molecules through the bloodstream towards their biological targets [53]. Within this context, it is necessary to investigate the affinity of bioactive compounds for albumins; such binding may differentiate the properties of the bioactive compound or provide novel transportation pathways or novel mechanisms of action [56]. For this reason, the interaction of AuNRs@PEG and AuNRs@PEG@NAP with HSA and its homologue BSA was studied by fluorescence emission quenching experiments (Figure S7 and Figure 8, respectively). When excitation at 295 nm was applied to the initial solutions of HSA and BSA, these solutions exhibited an intense fluorescence emission band at 337–339 nm which is attributed to tryptophan residues, i.e., tryptophan-214 in HSA, and tryptophan-134 and -212 in BSA [53]. The addition of AuNRs@PEG and AuNRs@PEG@NAP at increasing amounts into the albumins solutions resulted in the quenching of the respective band which was much more intense in the presence of AuNRs@PEG@NAP (up to 65.5% of the initial fluorescence of HSA, Figure 9 and Table 4). The inner-filter effect was also checked (Equation (S8)) [57] and it did not affect the measurements.

The quenching constants (k_q_) of the interaction of AuNRs@PEG and AuNRs@PEG@NAP with the SAs were determined with the Stern–Volmer quenching equation (Equations (S4) and (S5) [53,58]) where the fluorescence lifetime of tryptophan in SA is taken as τ_o_ = 10^−8^ s [58] and from the corresponding Stern–Volmer plots (Figures S8 and S9). The obtained k_q_ values (Table 4) may indicate the existence of a static quenching mechanism, verifying thus the interaction of the complexes with SAs.

The binding constants (K) of AuNRs@PEG and AuNRs@PEG@NAP with the SAs were determined from the Scatchard equation (Equation (S9)) [53] and the corresponding Scatchard plots (Figures S10 and S11). AuNRs@PEG@NAP presents a significantly higher (two to three times) affinity for both albumins than AuNRs@PEG (Table 4). The affinity of AuNRs@PEG for albumins is comparable with other gold nanoparticles [59,60] and gold nanoclusters [61]. A comparison of the constants of AuNRs@PEG@NAP with those of the reported metal–naproxen complexes is not possible due to their different concentration expression (the values of the complexes are expressed in the molar scale). Taken into consideration the molecular mass of NAP (230.2), its corresponding constants may also be expressed in mg/mL (Table 4) and, especially the value of K_HSA_, are much higher than those of AuNRs@PEG@NAP. The binding of AuNRs@PEG@NAP to albumins is reversible and relatively tight when compared to previously reported carriers of naproxen [43,44].

### 2.6. Evaluation of Cytotoxicity

In order to evaluate the biological effect of AuNRs@PEG and AuNRs@PEG@NAP, cytotoxicity studies were performed on two breast cancer cell lines: the low metastatic MCF-7 cells, and the more aggressive MDA-MB-231 cells. More specifically, AuNRs@PEG and AuNRs@PEG@NAP were used at a concentration of 1, 50, and 200 μg/mL and the cells’ viability was subsequently determined.

**Figure 10:**
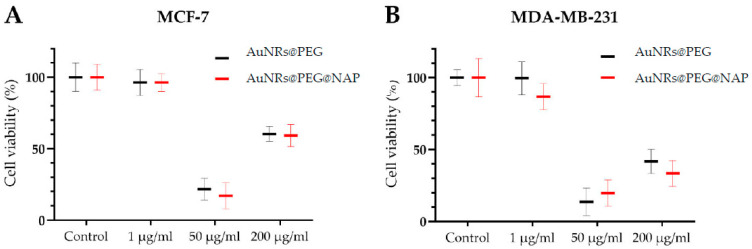
Cell viability of the breast cancer cells MCF-7 (**A**) and MDA-MB-231 (**B**) following treatments with different concentrations of AuNRs@PEG and AuNRs@PEG@NAP. Percentage is expressed relative to the non-treated cells (control) ± SD of experiments in triplicate.

Based on the results, the 1 μg/mL concentration of both AuNRs@PEG and AuNRs@PEG@NAP showed a slight, non-significant decrease of ~5% in cell viability in the MCF-7 cells. Similarly, AuNRs@PEG had the same effect in MDA-MB-231, whereas AuNRs@PEG@NAP exhibited a higher but not statistical decrease of ~13% in cell survival (Figure 10). Notably, the 50 μg/mL concentration showed a marked decrease in cell survival for AuNRs@PEG and AuNRs@PEG@NAP. At the higher concentration of 200 μg/mL, a significant decrease in cell viability for both AuNRs@PEG and AuNRs@PEG@NAP was also noted. The different cytotoxicity values between these two concentrations may be due to a different inhibitory mechanism or a stimulatory effect in cell growth at higher concentrations. Moreover, at 200 μg/mL the decrease in cell survival was more prominent on the aggressive MDA-MB-231 cells, where AuNRs@PEG decreased cell survival by ca 60% and AuNRs@PEG@NAP by ca 75%. In contrast, both AuNRs@PEG and AuNRs@PEG@NAP on the MCF-7 cells showed a decrease of ca 40%. Overall, both AuNRs@PEG and AuNRs@PEG@NAP exerted a similar effect on cell survival, with higher concentrations resulting in significant effects.

## 3. Experimental

### 3.1. Materials–Intrumentation–Physical Measurements

All the chemicals used and all solvents were of reagent grade and were used as purchased from commercial sources: NAP, CT DNA, EB, BSA, HSA, trisodium citrate, NaCl, NaBH4 (99%), Poly(ethylene glycol) methyl ether thiol (mPEG-SH, M_n_ = 6000), CH_3_COOH, and CH_3_COONa from Sigma-Aldrich Co; ascorbic acid (99.5%), cetyltrimethylammonium bromide (CTAB, 99%), sodium oleate (NaOL, 99%), HAuCl_4_·xH_2_O (99.999%, where x was estimated as 3), AgNO3 (99.9995%) from Alfa Aesar; Tris base, EDTA disodium salt dehydrate, loading buffer and H_2_O_2_ (30% *w*/*v*) from PanReac Applichem; supercoiled circular pBR322 plasmid DNA from New England Bioline; and all solvents from Chemlab.

The TEM study was performed utilizing a FEI CM20 TEM operating at 200 kV. TEM specimens were prepared by drop-casting a 3 μL droplet of AuNRs nanoparticles suspension on a carbon-coated Cu TEM grid. The size of the particles were determined by ‘‘manual counting’’ using the ImageJ (v. 1.54d) software (https://imagej.net, accessed on 4 February 2023).

For the interaction of AuNRs@PEG and AuNRs@PEG@NAP with biomacromolecules, different buffer solutions were prepared: (i) buffer solution with pH = 7 (bspH7) containing 150 mM NaCl and 15 mM trisodium citrate where the pH value of 7.0 was adjusted by HCl_(aq)_, (ii) Tris buffer (25 μM, pH 6.8) and (iii) buffer solution with pH = 4 (bspH4) containing 100 mM CH_3_COOH and 25 mM CH_3_COONa where the pH value of 7.0 was adjusted by HCl_(aq)_.

The DNA stock solution was prepared by dilution of CT DNA to a buffer solution of pH 7.0 (bspH7) followed by stirring at 4 °C and it was kept at 4 °C for no longer than two weeks. The stock solution of CT DNA gave a ratio of UV absorbance at 260 and 280 nm (A_260_/A_280_) in the range of 1.85–1.90, indicating that the DNA was sufficiently free of protein contamination [62]. The DNA concentration was determined by the UV absorbance at 260 nm after 1:20 dilution using ε = 6600 M^−1^ cm^−1^ [63]. UV-visible (UV-vis) spectra were recorded on a Hitachi U-2001 dual-beam spectrophotometer. The fluorescence emission spectra were recorded in solution on a Hitachi F-7000 fluorescence spectrophotometer.

### 3.2. Preparation of AuNRs@PEG and AuNRsPEG@NAP

The synthetic protocol of the seed solution used for the AuNRs growth is the following: 5 mL of HAuCl_4_ (0.5 mM) mixed with 5 mL CTAB (0.2 M), while an aqueous solution (1 mL) of 0.01 M NaBH_4_ was injected to the previous solution under vigorous stirring. The color of the solution change from yellow to brown-yellow and the stirring was terminated after 2 min. The seed solution was kept at RT for 30 min before use.

To prepare the growth solution, 7.0 g CTAB and 1.234 g NaOL were dissolved in a 1 L flask containing 250 mL of warm water (~50 °C). The solution was left to cool down and then an aqueous solution of AgNO_3_ (24 mL, 4 mM) was added and the mixture was kept without stirring for 15 min. Subsequently, 250 mL of HAuCl_4_ (1 mM) was added and the solution was stirred for about 1.5 h in order to become colorless. After that, 3 mL HCl (12.1 M) were used to adjust the pH value close to 1.6. Fifteen minutes later, 1.25 mL ascorbic acid (0.064 Μ) was added under vigorous stirring. Finally, 400 μL of seed solution was added in the resulted mixture and it was left undisturbed at 30 °C overnight in order to form the AuNRs. In order remove the excess of CTAB, the AuNRs were centrifuged at 10,000 rpm (12,298× *g*) (three times) and the supernatant was discarded while each time the pellet at the bottom of the falcon was redispersed in 15 mL H_2_O.

An amount of 30 mg of mPEG-SH was added in 10 mL of the previously prepared AuNRs solution (O.D. = 2.9, [Au] = 260 μg/mL), which was kept at 29 °C to avoid CTAB crystallization and the resulted solution was stirred for 24 h. The final product was isolated with centrifugation at 5900 rpm (4281× *g*) for 20 min followed by the removal of the supernatant (unreacted mPEG-SH) and redispersed in 10 mL H_2_O.

For loading the AuNRs@PEG with NSAID NAP, a clear colorless solution of 4 mg of NAP in 2 mL of chloroform was added in the previously prepared solution (10 mL) of AuNRs@PEG (O.D. = 2.4, [Au] = 239 μg/mL) and the mixture was treated in the ultrasound water bath for about 20 min, resulting a pale-pink emulsion. The chloroform was further evaporated by increasing the temperature in the water bath and the final clear solution was left undisturbed to cool at room temperature. The final AuNRs@PEG@NAP (O.D. = 2.32, [Au] = 197 μg/mL) in the form of supernatant was collected carefully by centrifuging the resulting solution for 5 min at 3900 rpm (1920× *g*) and was further lyophilized for the DNA-binding studies.

### 3.3. Study of the Interaction of the AuNRs with Biomacromolecules

Lyophilized AuNRs@PEG and AuNRs@PEG@NAP were initially dissolved in a buffer solution of pH = 4 (100 mM CH_3_COOH and 25 mM CH_3_COONa) at a concentration of 0.25 mg/mL. CT DNA and the albumins were dissolved in a buffer solution of pH = 7 (150 mM NaCl and 15 mM trisodium citrate).

The interaction of AuNRs@PEG and AuNRs@PEG@NAP with CT DNA was examined thoroughly by UV-vis spectroscopy for three different temperatures (291 K, 300 K, and 310 K), and via competitive studies with EB by fluorescence emission spectroscopy. The ability of AuNRs@PEG and AuNRs@PEG@NAP to cleave pDNA was studied by gel electrophoresis. The albumin (BSA or HSA) binding was studied through tryptophan fluorescence quenching experiments. All the specific protocols and relevant equations involved in the in vitro study of the interaction of the AuNRs@PEG and AuNRs@PEG@NAP with biomacromolecules are presented in the Supporting Information file (Sections S1–S3).

### 3.4. Drug-Release Protocol

The emission characterization of AuNRs@PEG@NAP was recorded on a Cary Eclipse emission spectrophotometer. The experimental protocol used for monitoring the drug release behavior was the following: 1 mg of AuNRs@PEG@NAP was dispersed in 5 mL of two buffered solutions (pH = 4.2, 6.8) under stirring at room temperature, achieving final concentration of 0.2 mg/mL. At predetermined time intervals, the solution was transferred in a cuvette and the emission intensity of NAP at 357 nm was recorded. Cumulative release (%) was given in Equation (2):

(2) $$\mathrm{Cumulative}\mathrm{release}\;(\%)=\frac{W_{t}}{W_{drug}}\times 100$$

where *W_t_* and *W_drug_* denote the weights of drug released from the hybrid material at time *t*, and the total amount of loaded drug, respectively. From the calibration curve of the emission intensity of NAP at 354 nm (Figure S3), the percentage of the drug loading can also be calculated as: 1 mg of the loaded material was dispersed in 5 mL of buffered solution giving 0.2 mg/mL final concentration. From the maximum emission intensity (184 a.u.) of NAP and the calibration curve, the quantity of naproxen in this dispersion is calculated at 0.022 mg/mL. As this value corresponds to 0.2 mg of the studied material, the loading is calculated at 112.5 mg/g.

### 3.5. Cell Culture

The breast cancer cell lines MCF-7 and MDA-MB-231 were obtained from the American Type Culture Collection (ATCC). The cells were cultured in a humidified 95% air/5% CO_2_ incubator at 37 °C in Dulbecco’s Modified Eagle’s Medium (DMEM), supplemented with 10% (*v*/*v*) fetal bovine serum (FBS), 1 mM sodium pyruvate, 2 mM L-glutamine, and 100 IU/mL penicillin.

### 3.6. Cytotoxicity Studies

MCF-7 and MDA-MB-231 cells were seeded on 12-well plates at a density of 5 × 10^4^ and cultured to 70% confluency. The synthesized compounds were dissolved in sterile ddH_2_O, and appropriate serial dilutions were prepared for further use. Following an overnight starvation in serum-free medium, the cells were incubated with AuNRs@PEG and AuNRs@PEG@NAP in different concentrations (1, 50, and 200 μg/mL) for 24 h. The adherent cells were subsequently harvested and counted manually, and the cytotoxicity was estimated as a percentage of living cells relative to non-treated control cells ± standard deviation (SD) of experiments in triplicate. The graphs were created with GraphPad Prism 8.

## 4. Conclusions

A facile experimental protocol for the synthesis of (PEGylated) gold nanorods (AuNRs@PEG) with AR = 6.0 is presented as well as an effective drug-loading procedure using non-steroidal anti-inflammatory drug (NSAID) naproxen (NAP). Drug release studies revealed a pH-sensitive behavior for AuNRs@PEG@NAP while the PEG content seems to determine the rate of release with a fast release up to a maximum of 24% and 33% NAP within the first 180 min at pH = 4.2 and 6.8, respectively.

The interaction of AuNRs with CT DNA was studied by UV-vis spectroscopy at three different temperatures and different interaction modes were concluded for each AuNR. More specifically, AuNRs@PEG may interact with CT DNA via van der Waals forces and hydrogen bonds on the external surface (groove-binding), while for AuNRs@PEG@NAP, π–π stacking interactions may be developed with CT DNA resulting in the possible intercalation of the naproxen moiety in-between DNA bases, which was further confirmed via its ability to displace the classical intercalator ethidium bromide from the EB–DNA adduct. The AuNRs may induce the moderate cleavage to pBR322 plasmid DNA at a rather high concentration (0.25 mg/mL) and AuNRs@PEG@NAP (48% of DNA cleavage) is more active than AuNRs@PEG.

The AuNRs studied herein showed a noteworthy affinity for bovine and human serum albumins and may become reversibly and tightly bound to them. In most studies, the affinity of the AuNR loaded with naproxen (AuNRs@PEG@NAP) for the albumins presents two to three times higher than that of AuNRs@PEG. The binding constants of both AuNRs studied herein to biomacromolecules are comparable with other reported similar systems including gold nanoparticles, gold nanoclusters, and carriers of naproxen (such as carbon nanotubes and spin-crossover nanoparticles), but slightly lower than most metal–naproxen complexes reported. Both AuNRs@PEG and AuNRs@PEG@NAP exerted similar cytotoxic effects against MCF-7 cells and MDA-MB-231 cancer cells and were more active against MDA-MB-231 cells. The exploration of loading/release mechanisms and DNA-binding studies for the case of other NSAID and anticancer drugs as well as the role of the molecular weight of mPEG-SH derivatives in the loading/release mechanism is under further study.

## Figures and Tables

**Table 1:** UV-vis spectral features of the interaction of AuNRs@PEG and AuNRs@PEG@NAP with CT DNA at 18 °C. UV band (λ in nm) (percentage of the observed hyper-/hypo-chromism (ΔA/A_0_, in %), blue/red shift of the λ_max_ (Δλ, in nm)) and DNA-binding constants (K_b_, in M^−1^).

Compound	λ (nm) (ΔA/A_ο_ (%) ^a^, Δλ (nm) ^b^)	K_b_ (M^−1^)
AuNRs@PEG	263 (−1 ^a^, 0 ^b^); 525 (−9, +5)	8.84 (±0.45) × 10^4^
AuNRs@PEG@NAP	267 (+10, 0); 319 (−9, +2); 530 (−10, +1)	5.25 (±0.07) × 10^4^
NAP [38,39]	325 (+22, +2)	2.67 (±0.22) × 10^4^

^a^ “+” denotes hyperchromism, “–” denotes hypochromism. ^b^ “+” denotes red shift, “–” denotes blue shift.

**Table 2:** Thermodynamic parameters of AuNRs@PEG and AuNRs@PEG@NAP for the interaction with CT DNA at different temperatures (291 K, 300 K, and 310 K).

Compound	T (K)	K_b_ (M^−1^)	ΔG (kcal M^−1^)	ΔH (kcal M^−1^)	ΔS (cal M^−1^ K^−1^)
AuNRs@PEG	291	8.84 (±0.45) × 10^4^	−2.861		
523 nm	300	3.28 (±0.07) × 10^4^	−2.692	−5.374	−8.74
	310	2.38 (±0.04) × 10^4^	−2.696		
AuNRs@PEG@NAP	291	5.25 (±0.07) × 10^4^	−2.729		
323 nm	300	8.85 (±0.17) × 10^4^	−2.949	+4.618	+25.24
	310	16.20 (±0.18) × 10^4^	−3.209		

**Table 3:** Fluorescence features of the EB–displacement studies: Percentage of EB–DNA fluorescence quenching (ΔI/Io), Stern–Volmer constants (K_SV_), and quenching constants of the EB–DNA fluorescence (k_q_) for AuNRs@PEG and AuNRs@PEG@NAP.

Compound	ΔI/Io (%)	K_SV_ ((mg/mL)^−1^)	k_q_ ((mg/mL)^−1^ s^−1^)
AuNRs@PEG	34.0	10.6 ± 0.4	4.59 (±0.18) × 10^8^
AuNRs@PEG@NAP	53.4	17.9 ± 0.3	7.79 (±0.15) × 10^8^
NAP [38,39]	82.0	1.47 (±0.04) × 10^5 a^ 639.1 ± 17.3	6.39 (±0.17) × 10^12 b^ 2.77 (±0.07) × 10^10^

^a^ expressed in M^−1^. ^b^ expressed in M^−1^s^−1^.

**Table 4:** Quenching of the SA fluorescence (ΔΙ/Ιο, %), Stern–Volmer constants (K_SV_) SA-quenching constants (k_q_) and SA-binding constants (K) for AuNRs@PEG and AuNRs@PEG@NAP.

Compound	ΔI/Io (%)	K_SV_ ((mg/mL)^−1^)	k_q_ ((mg/mL)^−1^ s^−1^)	K ((mg/mL)^−1^)	n
BSA					
AuNRs@PEG	30.8	9.33 ± 0.33	9.33 (±0.33) × 10^8^	10.10 ± 0.65	0.94
AuNRs@PEG@NAP	48.9	19.8 ± 0.4	1.98 (±0.04) × 10^9^	18.3 ± 0.69	0.96
NAP [38,39]	24.0	1.18 (±0.06) × 10^4 a^ 51.3 ± 2.6	1.18 (±0.06) × 10^12 b^ 5.13 (±0.26) × 10^9^	5.35 (±0.42) × 10^3 a^ 26.3 ± 1.8	2.14
**HSA**					
AuNRs@PEG	25.4	7.12 ± 0.27	7.12 (±0.27) × 10^8^	10.25 ± 0.38	0.80
AuNRs@PEG@NAP	65.5	38.2 ± 1.3	3.82 (±0.13) × 10^9^	29.49 ± 3.14	1.03
NAP [38,39]	22.7	1.24 (±0.09) × 10^4 a^ 53.9 ± 3.9	1.24 (±0.09) × 10^12 b^ 5.39 (±0.04) × 10^9^	3.27 (±0.30) × 10^4 a^ 142.17 ± 13.04	0.43

^a^ expressed in M^−1^. ^b^ expressed in M^−1^ s^−1^.

## Data Availability

Not applicable.

## Supplementary Materials

The following supporting information can be downloaded at: https://www.mdpi.com/article/10.3390/molecules28093780/s1, Figure S1: Gaussian distributions of sizes of AuNRs; Figure S2: Comparison between the emission spectra of the free NAP solution (red line, λ_max,em_ = 357 nm) and AuNRs@PEG@NAP (λ_max,em_ = 359 nm); Figure S3: Calibration line of the emission intensity of NAP at 357 nm as a function of the concentration of NAP; Figure S4: Plot of $\frac{\left[DNA\right]}{{(\varepsilon }_{A}-\varepsilon_{f})}$ versus [DNA] for the AuNRs; Figure S5: Fluorescence emission spectra (λ_exc._ = 540 nm) for EB–DNA adduct ([EB] = 20 μM, [DNA] = 26 μM) in buffer solution in the absence and presence of increasing amounts of AuNRs@PEG; Figure S6: Stern–Volmer quenching plot of EB–DNA fluorescence for the AuNRs; Figure S7: Fluorescence emission spectra (λ_excitation_ = 295 nm) for HSA ([HSA] = 3 μM) in buffer solution in the absence and presence of increasing amounts of the AuNRs; Figure S8: Stern–Volmer quenching plot of BSA fluorescence for the AuNRs; Figure S9: Stern–Volmer quenching plot of HSA fluorescence for the AuNRs; Figure S10: Scatchard plot of BSA for the AuNRs; Figure S11: Scatchard plot of HSA for the AuNRs. Sections S1–S3: Protocols, procedures, and equations regarding interaction studies with CT DNA (S1), DNA cleavage experiments (S2) and albumin-binding studies (S3) [40,53,54,57,58,64].

## Abbreviations

BSA = bovine serum albumin; bspH4 = buffer solution with pH 4; bspH7 = buffer solution with pH 7; CT = calf-thymus; ds = double-stranded nick; EB = ethidium bromide; HSA = human serum albumin; K = SA-binding constant; K_b_ = DNA-binding constant; k_q_ = quenching constant; K_SV_ = Stern–Volmer constant; NAP = naproxen; NP = nanoparticle; NR = nanorod; NSAID = non-steroidal anti-inflammatory drug; pDNA = pBR322 plasmid DNA; PEG = poly(ethylene glycol); SA = serum albumin; ss = single-stranded nick.
