# Supplementary material for: pH-Sensitive Gold Nanorods for Non-Steroidal Anti-Inflammatory Drugs (NSAIDs) Delivery and DNA-Binding Studies
Source: Molecules. 2023 Apr 27;28(9):3780. doi: 10.3390/molecules28093780 (PMC10179929; doi:10.3390/molecules28093780)
Supplement: Supplementary file 1 [file molecules-28-03780-s001.zip › molecules-2345556-supplementary.pdf]

pH-sensitive Gold Nanorods for Non-Steroidal Anti-Inflammatory Drugs (NSAIDs) Delivery and DNA-binding studies

Eleni Zygouri [1], Vlasoula Bekiari[2], Georgios Malis [3], Nikos K. Karamanos [4,5], Christos Koutsakis [4], George Psomas [3,*], Vassilis Tangoulis [1,*]

[1] Department of Chemistry, Laboratory of Inorganic Chemistry, University of Patras, 26504 Patras, Greece; eleni0503zig@hotmail.com
[2] Department of Crop Science, University of Patras, 30200 Messolonghi, Greece; bbekiari@upatras.gr
[3] Department of General and Inorganic Chemistry, Faculty of Chemistry, Aristotle University of Thessaloniki, 54124 Thessaloniki, Greece; georgosmalis@gmail.com
[4] Biochemistry, Biochemical Analysis & Matrix Pathobiology Research Group, Laboratory of Biochemistry, Department of Chemistry, University of Patras, 26504 Patras, Greece; n.k.karamanos@upatras.gr (N.K.K.); ckoutsakis@upatras.gr (C.K.)
[5] Institute of Chemical Engineering Sciences (ICE-HT), Foundation for Research and Technology-Hellas (FORTH), Patras 26504, Greece
* Correspondence: gepsomas@chem.auth.gr (G.P.); vtango@upatras.gr (V.T.)

Supplementary Materials

INDEX

Figure S1. Gaussian distributions of sizes of AuNRs. 3

Figure S2. Comparison between the emission spectra of the free NAP solution (red line, $\lambda_{max,em}$ = 357 nm) and AuNRs@PEG@NAP ($\lambda_{max,em}$ = 359 nm) 3

Figure S3. Calibration line of the emission intensity of NAP at 357 nm as a function of the concentration of NAP 4

Figure S4. Plot of $[DNA]/(\varepsilon A - \varepsilon f)$ *versus* [DNA] for AuNRs@PEG and AuNRs@PEG@NAP. 5

Figure S5. Fluorescence emission spectra ($\lambda_{excitation}$ = 540 nm) for EB–DNA adduct ([EB] = 20 μM, [DNA] = 26 μM) in buffer solution (150 mM NaCl and 15 mM trisodium citrate at pH = 7.0) in the absence and presence of increasing amounts of AuNRs@PEG. The arrow shows the changes of intensity upon increasing amounts of AuNRs@PEG. 5

Figure S6. Stern-Volmer quenching plot of EB-DNA fluorescence for AuNRs@PEG and AuNRs@PEG@NAP. 6

Figure S7. Fluorescence emission spectra ($\lambda_{excitation}$ = 295 nm) for HSA ([HSA] = 3 μM) in buffer solution (150 mM NaCl and 15 mM trisodium citrate at pH 7.0) in the absence and presence of increasing amounts of AuNRs@PEG and AuNRs@PEG@NAP. The arrow shows the changes of intensity upon increasing amounts of AuNRs@PEG and AuNRs@PEG@NAP. 6

Figure S8. Stern-Volmer quenching plot of BSA fluorescence for AuNRs@PEG and AuNRs@PEG@NAP. 7

Figure S9. Stern-Volmer quenching plot of HSA fluorescence for AuNRs@PEG and AuNRs@PEG@NAP. 7

Figure S10. Scatchard plot of BSA for AuNRs@PEG (1) and AuNRs@PEG@NAP (2). 8

Figure S11. Scatchard plot of HSA for AuNRs@PEG (1) and AuNRs@PEG@NAP (2). 8

S1 Interaction studies with CT DNA 9

S1.1 Binding study with CT DNA by UV-vis spectroscopy 9

S1.2 Determination of the thermodynamic parameters 9

S1.3 EB-displacement studies 9

S2 DNA–cleavage experiments 10

S3 Albumin-binding studies 11

References 12

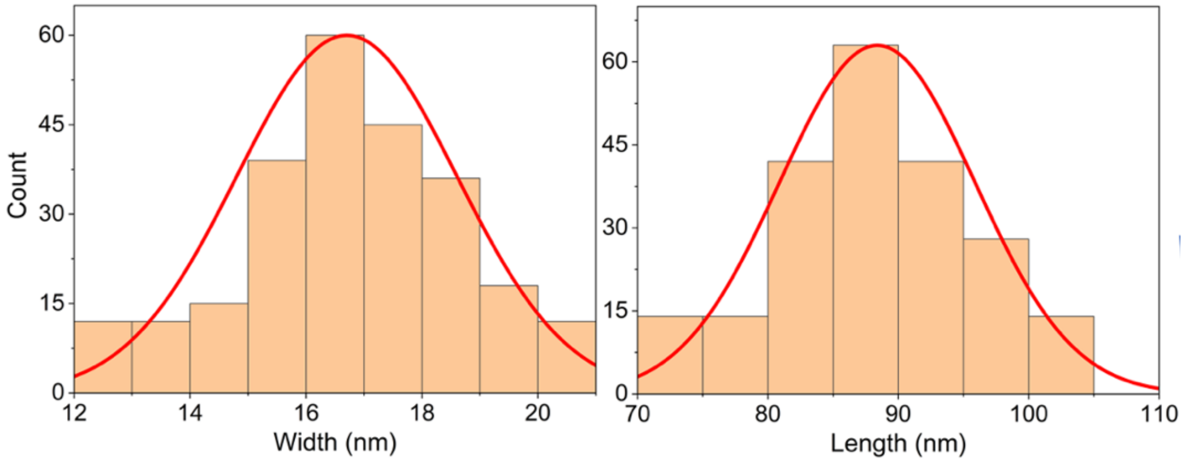

Figure S1. Gaussian distributions of sizes of AuNRs.

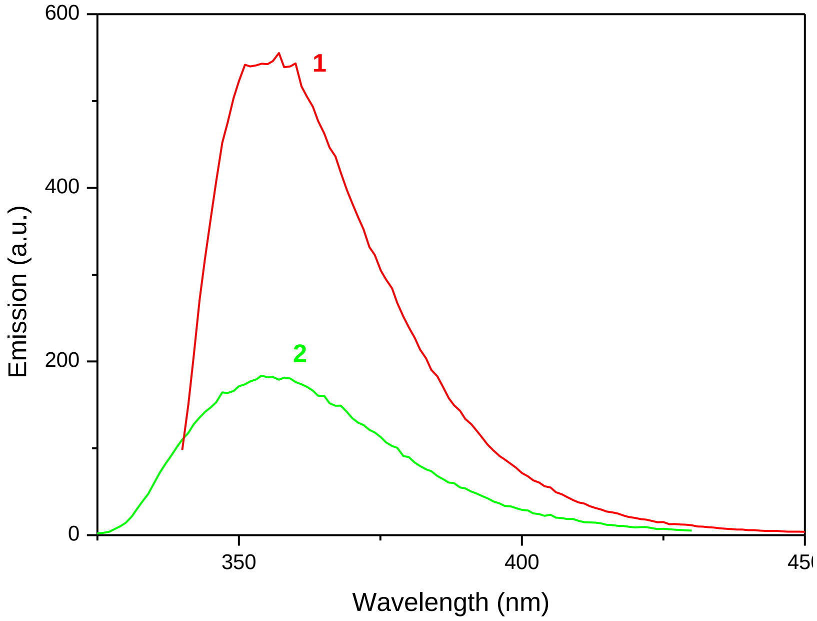

Figure S2. Comparison between the emission spectra of the free NAP solution (red line, $\lambda_{max,em}$ = 357 nm) and AuNRs@PEG@NAP ($\lambda_{max,em}$ = 359 nm).

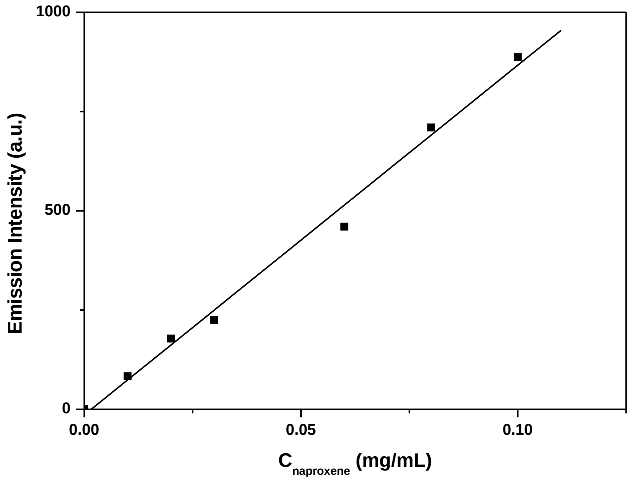

Figure S3. Calibration line of the emission intensity of NAP at 357 nm as a function of the concentration of NAP.

Linear Regression for Data1_B:
Y = A + B * X

Parameter	Value	Error
A	-14.24415	18.75409
B	8809.0301	339.18614

R	SD	N	P
0.99631	31.35015	7	<0.0001

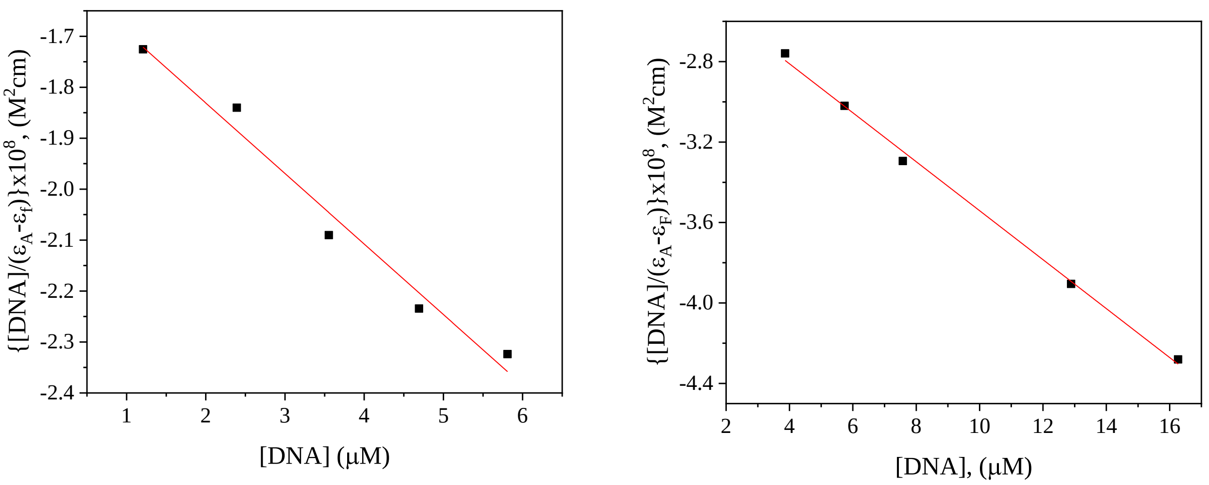

AuNRs@PEG AuNRs@PEG@NAP

Figure S4. Plot of $\frac{[DNA]}{(\varepsilon_A - \varepsilon_f)}$ *versus* [DNA] for AuNRs@PEG and AuNRs@PEG@NAP.

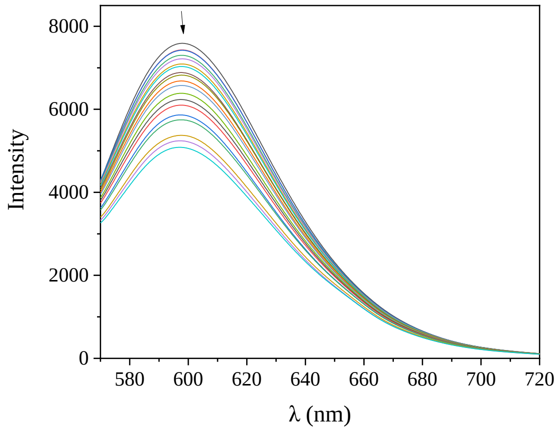

Figure S5. Fluorescence emission spectra ($\lambda_{excitation}$ = 540 nm) for EB–DNA adduct ([EB] = 20 μM, [DNA] = 26 μM) in buffer solution (150 mM NaCl and 15 mM trisodium citrate at pH = 7.0) in the absence and presence of increasing amounts of AuNRs@PEG. The arrow shows the changes of intensity upon increasing amounts of AuNRs@PEG.

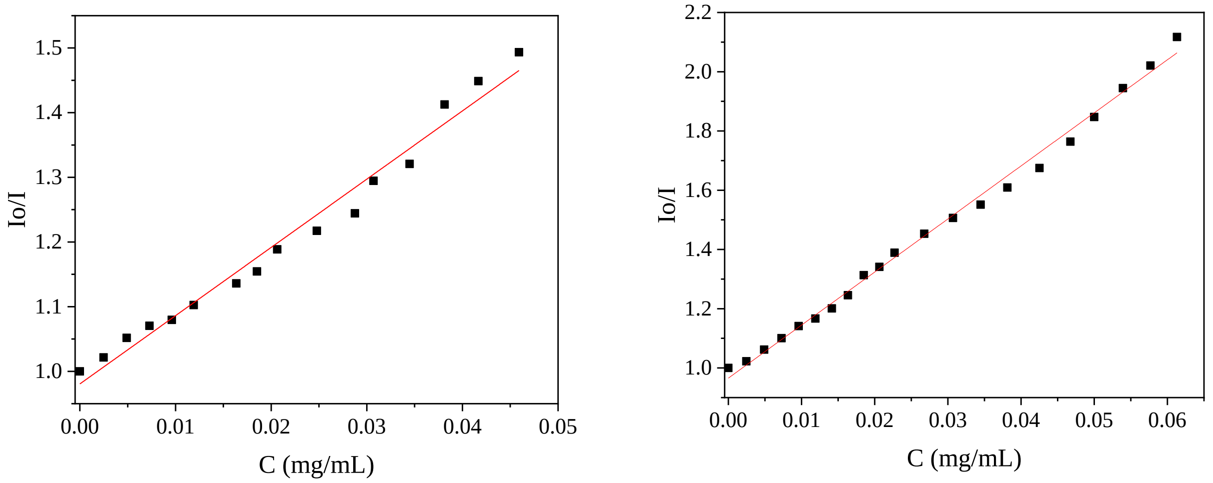

AuNRs@PEG AuNRs@PEG@NAP

Figure S6. Stern-Volmer quenching plot of EB-DNA fluorescence for AuNRs@PEG and AuNRs@PEG@NAP**.**

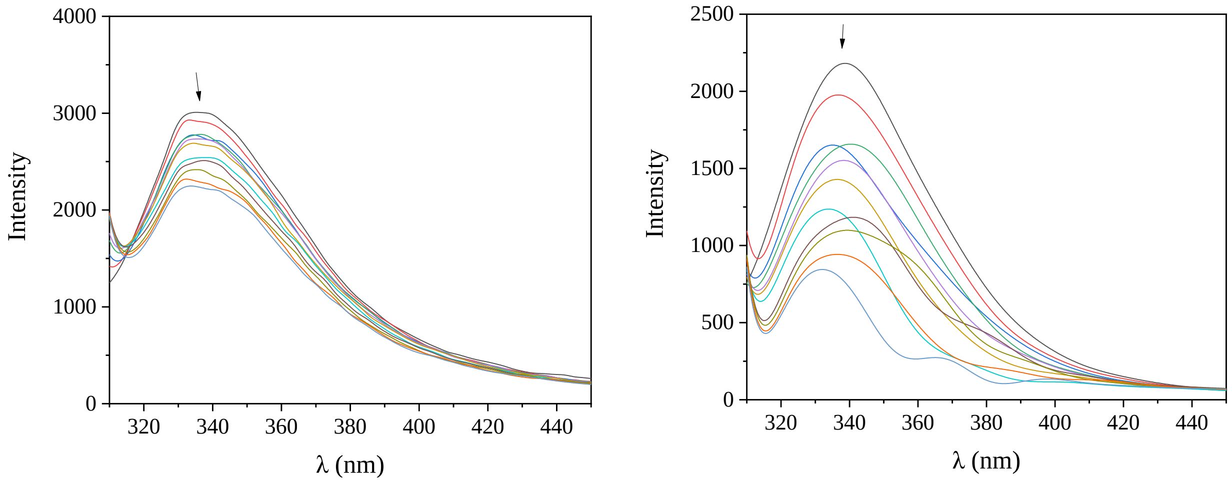

AuNRs@PEG AuNRs@PEG@NAP

Figure S7. Fluorescence emission spectra ($\lambda_{excitation}$ = 295 nm) for HSA ([HSA] = 3 μM) in buffer solution (150 mM NaCl and 15 mM trisodium citrate at pH 7.0) in the absence and presence of increasing amounts of AuNRs@PEG and AuNRs@PEG@NAP. The arrow shows the changes of intensity upon increasing amounts of AuNRs@PEG and AuNRs@PEG@NAP.

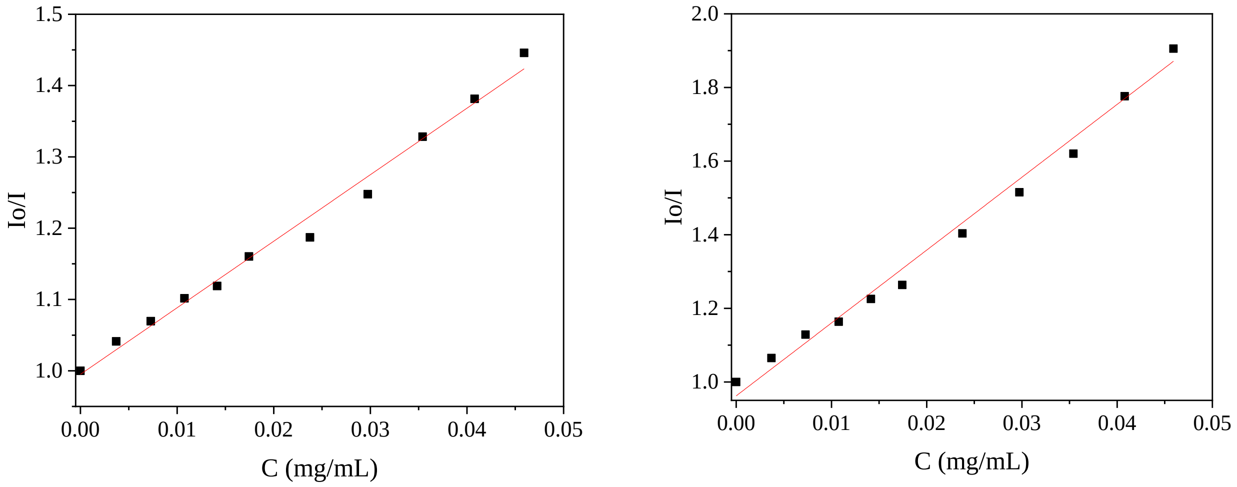

AuNRs@PEG AuNRs@PEG@NAP

Figure S8. Stern-Volmer quenching plot of BSA fluorescence for AuNRs@PEG and AuNRs@PEG@NAP.

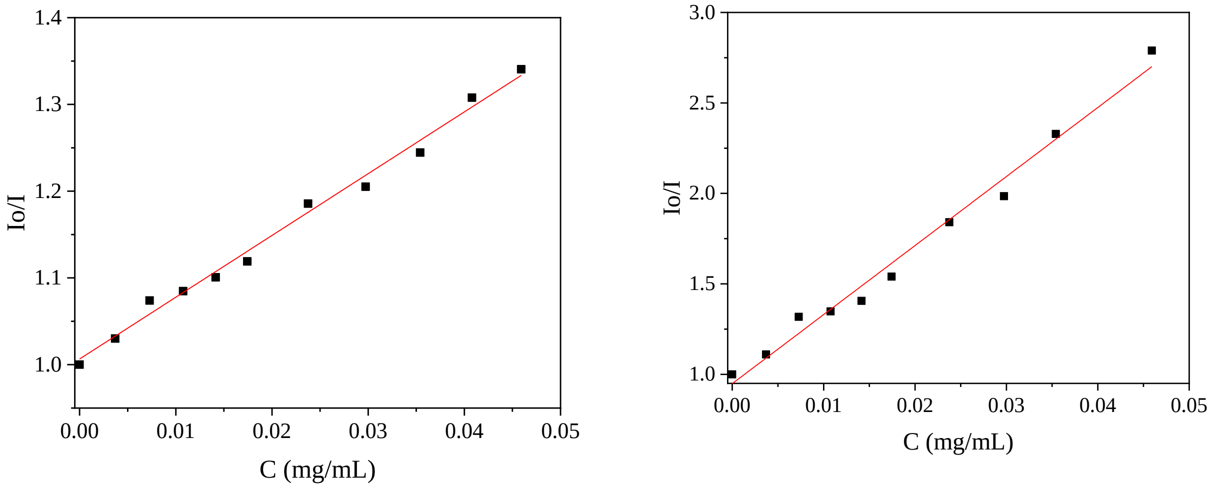

AuNRs@PEG AuNRs@PEG@NAP

Figure S9. Stern-Volmer quenching plot of HSA fluorescence for AuNRs@PEG and AuNRs@PEG@NAP.

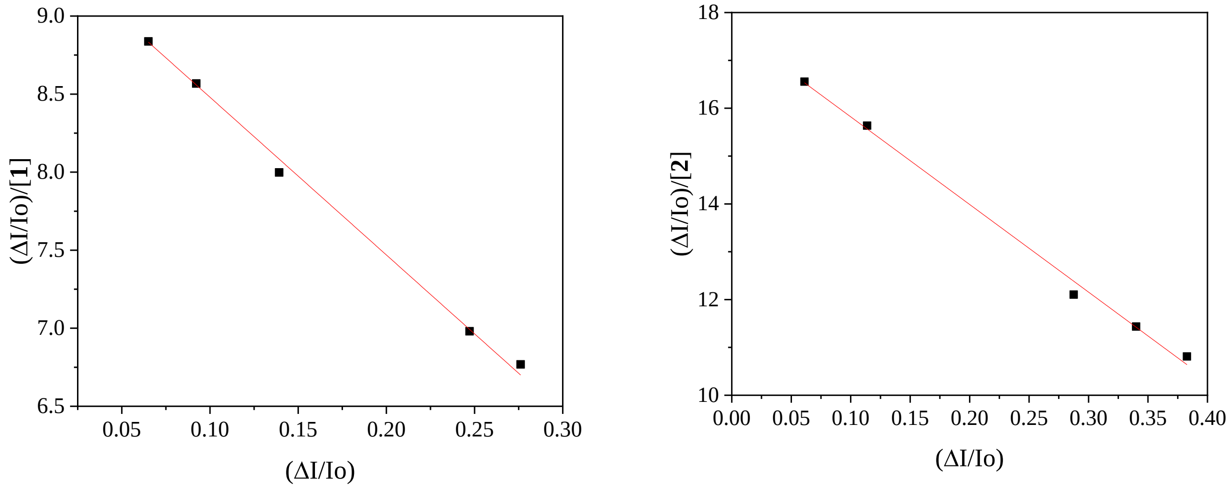

AuNRs@PEG AuNRs@PEG@NAP

Figure S10. Scatchard plot of BSA for AuNRs@PEG (**1**) and AuNRs@PEG@NAP (**2**).

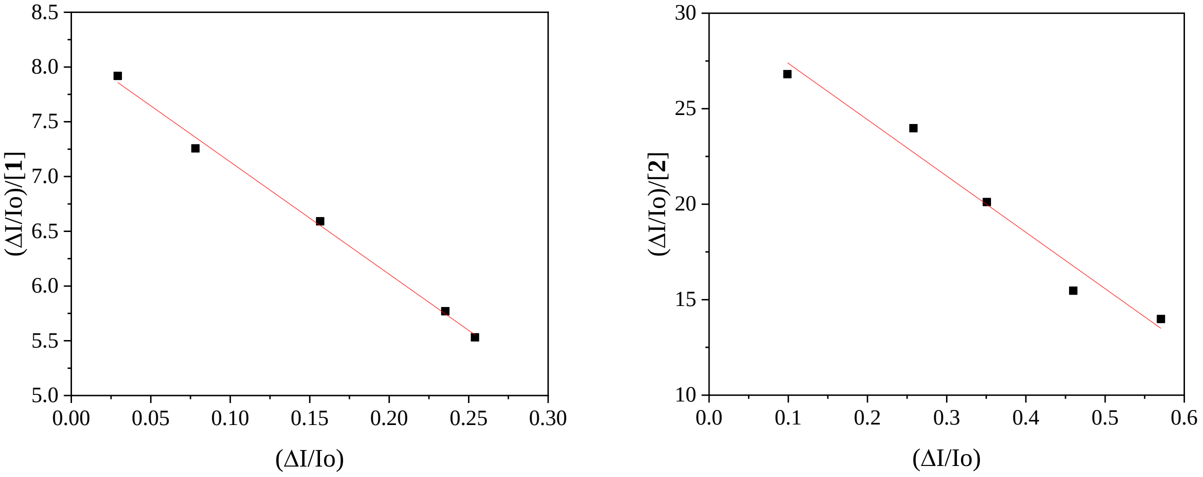

AuNRs@PEG AuNRs@PEG@NAP

Figure S11. Scatchard plot of HSA for AuNRs@PEG (**1**) and AuNRs@PEG@NAP (**2**).

S1 Interaction studies with CT DNA

The interaction of the AuNRs with CT DNA was studied by UV-vis spectroscopy at room temperature and variable-temperatures, and *via* competitive studies with EB by fluorescence emission spectroscopy.

S1.1 Binding study with CT DNA by UV-vis spectroscopy

The interaction of the AuNRs with CT DNA has been studied by UV-vis spectroscopy in order to investigate the possible binding modes to CT DNA and to calculate the DNA-binding constants (K_b). The K_b constants (in M^{-1}) were determined by the Wolfe-Shimer equation (eq. S1) [1] and the plots [DNA]/(ε_A-ε_f) *versus* [DNA] using the UV-vis spectra of the AuNRs (0.25 mg/mL) recorded in the presence of increasing concentrations of CT DNA. According to the Wolfe-Shimer equation (equation S1):

$$\frac{[\mathrm{DNA}]}{(\varepsilon_A - \varepsilon_f)} = \frac{[\mathrm{DNA}]}{(\varepsilon_b - \varepsilon_f)} + \frac{1}{K_b(\varepsilon_b - \varepsilon_f)} \qquad \text{(equation S1)}$$

where [DNA] is the concentration of DNA in base pairs, ε_A = A_{obsd}/[compound], ε_f = the extinction coefficient for the free AuNR, and ε_b = the extinction coefficient for the AuNR in the fully bound form. K_b is given by the ratio of slope to the y intercept in plots [DNA]/(ε_A-ε_f) *versus* [DNA].

S1.2 Determination of the thermodynamic parameters

In order to determine the thermodynamics parameters enthalpy change (ΔH), entropy change (ΔS) and ΔG, the DNA-binding constants of the AuNRs were determined for three different temperatures (291 K, 300 K and 310 K) as described in section S1.1 with equation S1 and the corresponding plots.

The enthalpy change (ΔH) and the entropy change (ΔS) can be calculated from the van't Hoff equation (equation S2):

$$\ln{(K_b)} = -\frac{\Delta \mathrm{H}}{RT} + \frac{\Delta S}{R} \qquad \text{(equation S2)}$$

where ΔH and ΔS can be determined from the plot of ln(K_b) *versus* (1/T), where – ΔH/R is the slope of the fitting line and ΔS/R is the intercept (R is the universal gas constant). In addition, ΔG may be obtained from the Gibb's-Helmholtz equation (equation S3):

$$\Delta G = \Delta H - T \cdot \Delta S \qquad \text{(equation S3)}$$

S1.3 EB-displacement studies

The competition of the AuNRs with EB was investigated by fluorescence emission spectroscopy in order to examine whether the AuNRs may displace EB from its DNA-EB adduct. The CT DNA-EB adduct was formed by pre-treating 20 μM EB and 26 μM CT DNA

in buffer (150 mM NaCl and 15 mM trisodium citrate at pH = 7.0). The possible displacement of EB by the AuNRs and subsequently their intercalating effect was studied by the stepwise addition of a certain amount of the solution of each AuNR into the solution of the CT DNA-EB adduct. The solutions were excited at 540 nm and the emission was monitored from 550-700 nm with λ_{max}= 592-595 nm and the effect of the addition of each AuNR to the CT-DNA EB solution was recorded. The AuNRs did not display any fluorescence emission bands at room temperature in solution or in the presence of CT DNA or EB under the same experimental conditions ($\lambda_{excitation}$ = 540 nm); therefore, the observed quenching of the EB-DNA solution may be attributed to the displacement of EB from its EB-DNA adduct.

The Stern-Volmer constants (K_{SV}, in M^{-1}) were calculated according by the linear Stern-Volmer equation (equation S4) [2] and the respective plots Io/I *versus* [AuNR].

$$\frac{Io}{I} = 1 + k_q \tau_0 [Q] = 1 + K_{SV}[Q] \qquad \text{(equation S4)}$$

where Io and I are the emission intensities of the EB-DNA solution in the absence and the presence of the AuNRs, respectively, τ_o= the average lifetime of the emitting system without the quencher and kq = the quenching constant. Taking τ_o = 23 ns as the fluorescence lifetime of the EB-DNA adduct [3], the quenching constants (k_q, in $M^{-1}s^{-1}$) of the AuNRs were calculated according to equation S5 [2]:

$$K_{SV} = k_q \tau_o \qquad \text{(equation S5)}$$

S2 DNA–cleavage experiments

The reaction mixtures (20 μL) containing supercoiled circular pBR322 DNA stock solution (Form I, 50 μM/base pair, ~500 ng), AuNRs, and Tris buffer (25 μM, pH 6.8) in Pyrex vials were incubated for 30 min at 37 °C, and was centrifuged under aerobic conditions at room temperature.

After addition of the gel–loading buffer [6X Orange DNA Loading Dye 10 mM Tris–HCl (pH 7.6), 0.15% orange G, 0.03% xylene cyanol FF, 60% glycerol, and 60 mM EDTA, by Fermentas], the reaction mixtures were loaded on a 1% agarose gel with EB staining. The electrophoresis tank was attached to a power supply at a constant current (65 V for 1 h). The gel was visualized by 312 nm UV transilluminator and photographed by an FB−PBC−34 camera vilberlourmat. Quantification of DNA−cleaving activities was performed by integration of the optical density as a function of the band area using the program "Image J" available at the site http://rsb.info.nih.gov/ij/download.html.

The ss% and ds% damages were calculated according to the equations S6 and S7:

$$ss\% = \frac{FormII}{(FormI + FormII + FormIII)} \times 100 \qquad \text{(equation S6)}$$

$$ds\% = \frac{FormIII}{(FormI + FormII + FormIII)} \times 100 \qquad \text{(equation S7)}$$

where, as Form II we consider Form II of each series minus Form II of the irradiated control DNA and as Form I, we consider Form I of each series. The amount of supercoiled DNA was multiplied by factor of 1.43 to account for reduced EB intercalation into supercoiled DNA [4].

S3 Albumin-binding studies

In order to investigate if the AuNRs can bind to carrier proteins like serum albumins, we carried out albumin-binding study by tryptophan fluorescence quenching experiments using bovine serum albumin (BSA, 3 μM) or human serum albumin (HSA, 3 μM) in buffer (containing 15 mM trisodium citrate and 150 mM NaCl at pH 7.0). The quenching of the emission intensity of tryptophan residues of BSA at 343 nm was monitored using the AuNRs as quenchers with increasing concentration [2]. The fluorescence emission spectra of the AuNRs were also recorded with λ_{ex} = 295 nm. No fluorescence emission band was recorded and thus it was not related to the AuNRs. The influence of the inner-filter effect [5] on the measurements was evaluated by equation S8.

$$I_{corr} = I_{meas} \times 10^{\frac{\varepsilon(\lambda_{exc})cd}{2}} \times 10^{\frac{\varepsilon(\lambda_{em})cd}{2}} \quad \text{(equation S8)}$$

where I_{corr} = corrected intensity, I_{meas} = the measured intensity, c = the concentration of the quencher, d = the cuvette (1 cm), $\varepsilon_{(\lambda exc)}$ and $\varepsilon_{(\lambda em)}$ = the ε of the quencher at the excitation and the emission wavelength, respectively, as calculated from the UV-vis spectra of the AuNRs [5].

The Stern-Volmer and Scatchard graphs were used to study the interaction of the compounds with serum albumins. According to Stern-Volmer quenching equation (eq. S2), where Io = the initial tryptophan fluorescence intensity of SA, I = the tryptophan fluorescence intensity of SA after the addition of the quencher, k_q = the quenching constant, K_{SV} = the Stern-Volmer constant, τ_o = the average lifetime of SA without the quencher, and, taking as fluorescence lifetime (τ_o) of tryptophan in SA at around 10^{-8} s [2], K_{SV} can be obtained by the slope of the diagram Io/I *versus* [AuNR] (Stern-Volmer plots), and subsequently the quenching (k_q) may be calculated from eq. S3.

From the Scatchard equation (equation S9):

$$\frac{\Delta I / Io}{[Q]} = nK - K\frac{\Delta I}{Io} \quad \text{(equation S9)}$$

where n is the number of binding sites per albumin and K is the SA-binding constant (K, in M^{-1}) is calculated from the slope in plots (ΔI/Io)/[AuNR] *versus* ΔI/Io and n is given by the ratio of y intercept to the slope [2,6].

References

1. Wolfe, A.; Shimer, G.H.; Meehan, T. Polycyclic Aromatic Hydrocarbons Physically Intercalate into Duplex Regions of Denatured DNA. *Biochemistry* **1987**, *26*, 6392–6396.
2. Lakowicz, J.R. Principles of Fluorescence Spectroscopy. *Principles of Fluorescence Spectroscopy* **2006**, 1–954.
3. Heller, D.P.; Greenstock, C.L. Fluorescence Lifetime Analysis of DNA Intercalated Ethidium Bromide and Quenching by Free Dye. *Biophys Chem* **1994**, *50*, 305–312.
4. Papastergiou, A.; Perontsis, S.; Gritzapis, P.; Koumbis, A.E.; Koffa, M.; Psomas, G.; Fylaktakidou, K.C. Evaluation of O-Alkyl and Aryl Sulfonyl Aromatic and Heteroaromatic Amidoximes as Novel Potent DNA Photo-Cleavers. *Photochemical and Photobiological Sciences* **2016**, *15*, 351–360.
5. Stella, L.; Capodilupo, A.L.; Bietti, M. A Reassessment of the Association between Azulene and [60]Fullerene. Possible Pitfalls in the Determination of Binding Constants through Fluorescence Spectroscopy. *Chemical Communications* **2008**, 4744–4746.
6. Wang, Y.-Q.; Zhang, H.-M.; Zhang, G.-C.; Tao, W.-H.; Tang, S.-H. Interaction of the Flavonoid Hesperidin with Bovine Serum Albumin: A Fluorescence Quenching Study. *J Lumin* **2007**, *126*, 211–218.
